# The COVID-19 pandemic response and its impact on post-corona health emergency and disaster risk management in Italy

**DOI:** 10.3389/fpubh.2022.1034196

**Published:** 2022-10-31

**Authors:** Alessandro Lamberti-Castronuovo, Emanuela Parotto, Francesco Della Corte, Ives Hubloue, Luca Ragazzoni, Martina Valente

**Affiliations:** ^1^CRIMEDIM-Center for Research and Training in Disaster Medicine, Humanitarian Aid and Global Health, Università del Piemonte Orientale, Novara, Italy; ^2^Department for Sustainable Development and Ecological Transition, Università del Piemonte Orientale, Vercelli, Italy; ^3^Department of Surgery DIDAS, Azienda Ospedale Università, Padova, Italy; ^4^Department of Translational Medicine, Università del Piemonte Orientale, Novara, Italy; ^5^Research Group on Emergency and Disaster Medicine, Vrije Universiteit Brussel, Brussels, Belgium

**Keywords:** COVID-19, Italy, H-EDRM, disaster response, qualitative research

## Abstract

**Background:**

The COVID-19 pandemic has profoundly impacted societies, influencing countries' Health Emergency and Disaster Risk Management (H-EDRM) systems. By taking Italy as a case study, this research aimed to investigate the response to the COVID-19 pandemic, focusing on the changes made to the existing H-EDRM system, with an emphasis on human resources, health service delivery, and logistics and the forward-looking strategies for the next health emergencies and disasters.

**Methods:**

We performed a retrospective observational case study using qualitative methodology. Data was collected *via* semi-structured interviews and analyzed considering the World Health Organization (WHO) H-EDRM framework. Multiple interviewees were selected to obtain a holistic perspective on the Italian response to COVID-19. Stakeholders from five different sectors (policy-making, hospital, primary care, third sector, lay community) from three of the most impacted Italian regions (Piemonte, Lombardia, and Veneto) were interviewed, for a total of 15 respondents.

**Results:**

Results on human resources revolved around the following main themes: personnel, training, occupational health, and multidisciplinary work; results on health service delivery encompassed the following main themes: public health, hospital, and primary care systems; results on logistics dealt with the following themes: infrastructures, supplies, transports, and communication channels. Lessons learned stressed on the importance of considering pragmatic disaster preparedness strategies and the need for cultural and structural reforms. Stakeholders mentioned several implications for the post-pandemic H-EDRM system in Italy.

**Conclusions:**

Findings highlight that the interconnection of sectors is key in overcoming pandemic-related challenges and for future disaster preparedness. The implications for the Italian H-EDRM system can inform advancements in disaster management in Italy and beyond.

## Introduction

In 2019, the World Health Organization (WHO) developed the Health Emergency and Disaster Risk Management (H-EDRM) framework, which provides guidance to countries and partners on developing capacities to reduce the risks and impacts of all types of emergencies and disasters including epidemics and pandemics. The H-EDRM framework comprises a set of guidelines based on multisectoral emergency and disaster management, capacities for implementing the international health regulations, health system building blocks, and good practices from regions, countries, and communities. It focuses mainly on the health sector, noting the need for collaboration with other sectors that make substantial contributions to reducing health risks and consequences of disasters (1).

The recent COVID-19 pandemic has exposed several shortcomings of countries' H-EDRM systems, particularly the lack of adequate disaster preparedness strategies, shedding light on a discrepancy between what is promoted by international frameworks and what happens in real life when a disaster occurs. In many cases, the response to the COVID-19 outbreak has not been uniform across the healthcare system or between health and other sectors, resulting in fragmented response strategies. Thus, several countries paid a high toll in terms of loss of life, economic repercussions, and increased poverty. This has been the case for Italy, which has been one of the largest epicenters globally, with almost 16 million cases and approximately 170 thousand deaths since the beginning of the pandemic (2).

The Italian national healthcare system (i.e., Sistema Sanitario Nazionale), a Beveridge-type healthcare system based on the principles of universal access and free healthcare, has faced increasing pressure during the pandemic. Years of fragmentation and decades of finance cuts, privatization, and deprivation of human and technical resources have restricted timely interventions and compromised a strong national coordination (3). Although cases have been registered across the entirety of the Italian territory, COVID-19 initial distribution was more sustained in the Northern and Central regions. Up until March 2020, the Lombardia region had experienced the heaviest burden, with over 40.000 detected cases, followed by the Veneto and Piemonte regions (4). Lombardia experienced the most consistent load of deaths, reaching a peak of more than 23.000 deaths 2 months after the beginning of the first wave. This is equivalent to an excess mortality of +118% compared to the average mortality rate in January-April 2015-2019 (5).

The most immediate challenge that Italy faced during the first wave was the overburdening of emergency services, with overcrowded emergency departments and rapidly saturated Intensive Care Units (ICUs) (6, 7). Hospitals were challenged on multiple fronts, including allocation of limited resources, infection prevention and control (IPC) measures, and adaptation of existing services to a rapidly evolving situation (8). The shortage of adequate personal protective equipment (PPE) has contributed to exposing healthcare workers (HCWs) to a high risk of contagion: at the beginning of 2020, HCWs represented 10% of Italy's confirmed COVID-19 cases (9).

The heavy impact of the COVID-19 pandemic was felt not only in hospital settings, but also at the community level. Italy was the first European country that introduced stringent lockdown measures, including restrictions on movement, closure of schools, and interruption of non-essential productive activities. Although the government implemented strategies to contain the outbreak (e.g., mass testing, case-detection, contact-tracing, isolation, and quarantine), these were managed by local public health authorities (i.e., Servizio Igiene e Sanità Pubblica, SISP), sub-regional entities under the control of the Local Health Authority (i.e., Azienda Sanitaria Locale, ASL), and were poorly integrated with the standard Primary Healthcare (PHC) system (10). PHC physicians were also overwhelmed and, as a result, additional units were established to reinforce territorial health services and grant continuity of operations for detection, assessment, rapid reporting, and active surveillance of suspected or confirmed cases. The so-called *Unità Speciali di Continuità Assistenziale* (USCA) were also poorly integrated within the PHC system, which resulted in a fragmented response at the community level (11).

Although the Italian response to the COVID-19 pandemic has shed light on an unprepared health system, unable to function when this was most needed, the experience of the COVID-19 pandemic has prompted the implementation of several disaster response strategies, which have ultimately resulted in a number of lessons learned and changes in the H-EDRM system. Understanding countries' response to COVID-19 can thus be an opportunity to learn about the best practices, measures taken, resolutions made, lessons learned, and future implications for the post-pandemic H-EDRM system.

In the attempt to examine previous disaster response mechanisms in Italy, it emerges that the body of scientific literature performing in-depth analyses on disaster management mechanisms is scant. The majority of the Italian disaster-related literature focuses on the Aquila earthquake in 2009 and of relevance is the analysis carried out by Alexander in 2010. According to Alexander (12), the main limitations characterizing the disaster response system in Italy were the fragmentation in the response apparatus and coordination mechanisms, the underestimation of the disaster preparedness and disaster risk reduction phases, and a top-down approach to emergency management (13). Notably, however, the fact that Italy is a disaster prone country with a long lasting experience in disaster response has not made Italy more prepared for the COVID-19 pandemic. There might be two explanations for this: first, the pandemic itself was an exceptional disaster both for its magnitude and consequences on the Italian health system, and second, there is a lack of an all-hazard approach to disaster management, where the disaster response strategy is able to respond to a broad variety of hazards.

To the best of our knowledge, there is little in-depth qualitative evidence on the Italian response to the COVID-19 pandemic. Capturing the lived experiences of stakeholders who had a prominent role during the pandemic, or were directly impacted by it, can contribute to a better understanding of the Italian disaster response strategy. Such a qualitative insight will inform the post-pandemic health system reforms and help to be better prepared for future crises. By taking Italy as a case study, this research aimed to investigate the response to the COVID-19 pandemic, focusing on the changes made to the existing H-EDRM system, with an emphasis on human resources, health service delivery, and logistics and the forward-looking strategies for the next health emergencies and disasters.

## Methods

We performed an in-depth retrospective qualitative study using semi-structured interviews to explore the perspectives of multiple stakeholders on the Italian response to the COVID-19 pandemic. The methods have been reported following the Consolidated Criteria for Reporting Qualitative Research (COREQ) (14).

### Research team and reflexivity

Semi-structured interviews were conducted by three researchers (ALC, EP, and MV) having previous experience with qualitative research and a global health educational background. All three interviewers participated in each interview, with one interviewer taking the lead in each interview.

### Study design

In this study, we adopted a qualitative case study design, namely a technique that allows researchers to explore a complex phenomenon within a specific context (15). We used purposive sampling to select participants that could provide rich, accurate and diversified data in relation to the research objective. We considered two criteria for the selection of respondents: (a) location: stakeholders belonging to the three most affected Italian regions (i.e., Piemonte, Lombardia, Veneto); (b) sector: stakeholders belonging to different sectors that could share their perspectives on the health response to the COVID-19 pandemic in Italy (i.e., policy-making, hospital, primary healthcare, third sector, lay community). These two criteria were combined and one stakeholder per each sector in each region was interviewed. Stakeholders were contacted *via* email. Information on the study objective, methodology and ethical implications were reported in the email. Upon confirmation of participation, stakeholders were proposed several time slots for the interview, and they chose the one fitting their schedules. Interviews were conducted online using Zoom [version: 5.10.4 (6592)].

### Data collection

A semi-structured interview guide made up of leading and probing questions (Supplementary materials 1, 2) was developed keeping in mind the objective of the study. The interview guide was divided in four sections investigating challenges, response strategies, lessons learned, and changes to the H-EDRM system. A specific focus was given to the following areas: human resources, health services delivery and logistics. The guide was piloted on a pool of fellow researchers, and it was adjusted following their feedback. The same interview guide was used to interview all respondents, with minor linguistic adaptations to address stakeholders from different sectors. Interviews were conducted from the second week of February 2022 to the second week of March 2022 and lasted from 1 to 1.5 h. After receiving consent from the respondents, interviews were audio recorded. Notes were taken during the interviews by interviewers not leading the session. Interviews were conducted in Italian; text segments reported in this article have been translated for publication purposes.

### Data analysis and reporting

Interviews were transcribed verbatim by using the Sonix software. Transcripts were manually checked to assess completeness and quality. Three researchers (ALC, EP, and MV) independently read all the transcripts to familiarize with the data. A codebook was developed by relying on the study objective and the concepts enunciated by the WHO H-EDRM framework. The codebook was used to deductively code the transcripts and it was kept flexible to be adapted in case new codes emerged inductively from the text. The analysis was performed in parallel by the three researchers. In particular, each researcher coded one specific thematic area (i.e., health workforce, health services delivery and logistics), and then the other two researchers independently checked the analysis on each area for consistency and validation. Any disagreement was resolved by confronting the team. Results have been summarized in a table format and are reported in the following section in written format. Translated quotes have been added to the text to enrich the study findings.

### Ethical considerations

The study was conducted according to the principles enunciated in the Declaration of Helsinki, and approved by the Ethics Committee of the A.O.U. “Maggiore della Carità” di Novara (Protocol 296/CE Study number CE 055/22) on April, 8th 2021. All participants were required to give oral informed consent prior to data collection. Sufficient details were provided at the beginning of the interview about the study aim and process. The data collected was anonymized and access to the data was restricted to the co-authors of this paper only.

### Disclosure

This study is a part of the WHO Kobe Center funded project “The COVID-19 pandemic response and its impact on post-Corona health emergency and disaster risk management” which involves Japan, Korea, Mongolia, Iran, Italy, Thailand, and the United States.

## Results

### Characteristics of the sample

Of the fifteen participants enrolled in this study, ten were males and five females. Nine of them held a managerial role in their professional sector, four of them had <5 years of experience, while the others had more than 9 years of experience. Interviewed medical doctors had been working in the healthcare sector for at least 15 years. Lay community stakeholders included three patients (two males and one female). All of them presented one or more chronic diseases; two of them (one female and one male) were affected by COVID-19 during the first and the second pandemic wave, respectively (Table 1) (for an overview of the study findings, refer to Supplementary materials 3–6).

**Table 1:** Demographic information for interview participants (ICU, Intensive Care Unit; NGO, Non-Governmental Organization; USCA, Special unit for continuity of care).

**Region**	**Policy-making**	**Hospital**	**Primary care**	**Third sector**	**Community member**
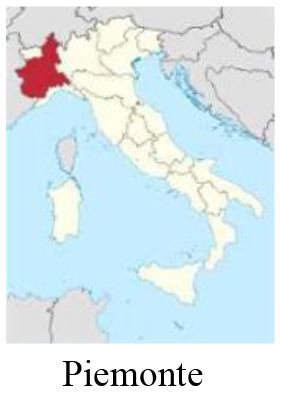	Role: mayor Years of experience (in the current role): 10 Sex: male Site: municipality among the most affected in Piemonte during the first COVID-19 pandemic wave	Role: hospital nurse bed manager Years of experience (in the current role): 12 Sex: female Site: second level hospital with a catchment area of 600.000–1.200.000 inhabitants	Role: USCA physician Years of experience (in the current role): 5 Sex: female Site: USCAs of North Piedmont region	Role: international NGO director Years of experience (in the current role): 12 Sex: male NGO mission: to contribute to the sustainable development of Africa by intervening in the health sector	Patient Sex and age: male, 75 years old Chronic diseases: diabetes, hypertrophic cardiomyopathy
					
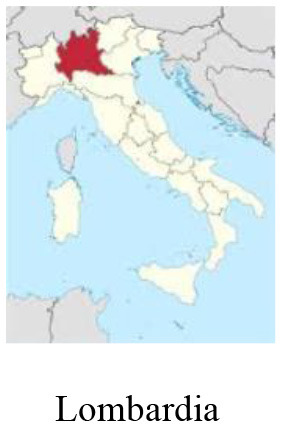	Role: mayor Years of experience (in the current role): 5 Sex: male Site: municipality among the most affected in Lombardia during the first COVID-19 pandemic wave	Role: head of Infectious Disease Unit Years of experience (in the current role): 14 Sex: male Site: university second level hospital with a catchment area: 600.000–1.200.000 inhabitants	Role: nursing home director Years of experience (in the current role): 23 Sex: male Site: nursing home with 130 patients	Role: nurse employed as Team Leader in an international NGO Years of experience (in the current role): 9 Sex: female NGO mission: to provide free medical treatment to the victims of war and poverty	Patient Sex and age: female, 42 years old Chronic diseases: asthma Other: mild COVID-19 disease during the puerperium
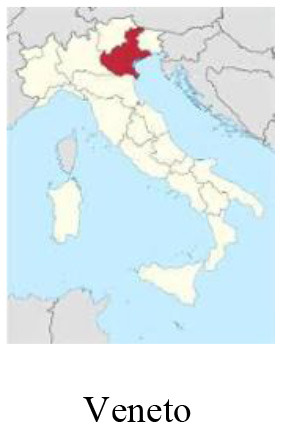	Role: mayor Years of experience (in the current role): 17 Sex: male Site: municipality among the most affected in Veneto during the first COVID-19 pandemic wave	Role: director of Anesthesia and Intensive Care Unit Years of experience (in the current role): 3 Sex: male Site: first level hospital with a catchment area: 150.000–300.000 inhabitants	Role: general practitioner Years of experience (in the current role): 2 Sex: female Site: city of approximately 200.000 inhabitants. Patients panel size: 1.600 patients	Role: head of International relations in an international NGO Sex: Male NGO mission: to promote and protect health in Africa	Patient Sex and age: male, 61 years old Chronic diseases: diabetes, multiple sclerosis, chronic ischemic heart disease, hypertension Other: hospitalization for severe COVID-19 disease with admission to ICU (length of stay: 30 days)

### Human resources

The following main themes emerged when examining human resources: (1) management of human resources; (2) education and training; (3) occupational health and safety; and (4) multisectoral and multidisciplinary collaboration.

#### Human resources management

Participants identified the shortage of HCWs as the main challenge experienced during the pandemic. This was described as a consequence of a chronic dearth of HCWs, both in hospitals and PHC centers. The pandemic worsened this shortage *via* the increased demand for staff, the infection of many HCWs, the suspension from work of those refusing vaccination, an initial low participation rate of those fearing contagion, and the absorption of non-hospital HCWs by hospitals. As a response, all deferrable procedures (e.g., follow-up visits for chronic conditions and elective surgery procedures) were interrupted in order to quickly reallocate HCWs to COVID-19 wards. New *ad hoc* employment contracts were developed to recruit additional HCWs among medical residents, free-lance HCWs, and recent medicine and nursing sciences graduates. HCWs were also recruited with the help of non-governmental organizations (NGOs): “*All staff members who had been employed abroad were reallocated to COVID-19 wards in their home countries”* (third sector, Lombardia). A relevant contribution was given by volunteers employed to support health facilities and municipalities. On the one hand, the involvement of volunteers was described as fundamental by some participants, because it reinforced health system resilience. On the other hand, stakeholders suggested not to rely on volunteers on a long-term basis, as this strategy would not be sustainable in case of protracted emergencies.

Participants mentioned low salaries, poor opportunities of career progression and excessive or inadequate distribution of workloads as other relevant difficulties encountered during the pandemic. The daily workload of HCWs has further increased when they had to take on significant administrative tasks: “A *bureaucratic-administrative* [resource] *was missing…doctors don't have to be administrative employees, they have to be doctors”* (hospital, Lombardia). Although the need to improve contractual measures was reported as an important lesson learned, no significant response strategy was reported in this regard except for a proposal of a law concerning a new employment system for PHC physicians, which aims at integrating them within the national health system, rather than independent free-lance workers.

Overall, interviewees described a chaotic organization of the workload, disorganized allocation of HCWs to different health services, lack of long-term contracts, and poor coordination among regional operation centers and local health facilities. The importance of having an adequate centralized management of human resources to guarantee coordination and integration of HCWs at multiple levels was a fundamental lesson learned.

#### Education and training

Interviewees pointed out the lack of adequately trained HCWs on the correct use of PPE and on general principles of disaster response, especially at the beginning of the pandemic. These challenges were experienced by hospitals as well as PHC centers. Young physicians and nurses were deployed to COVID-19-related emergency services without adequate training or assistance: “*They were literally thrown into Intensive Care Units and Emergency Departments...the residents had to learn on their own”* (third sector, Piedmont). The response strategies that were put in place were aimed at training recent graduates and HCWs who had to work in COVID-19 wards. These measures included on-line training courses and on-the-job training strategies on the use of PPE and the management of COVID-19 patients. In some cases, therapeutic protocols were established at a regional level to allow better coordination of health services. NGOs gave support to healthcare facilities by providing on-line or face-to-face courses concerning the use of PPE and the implementation of IPC measures: ”*We immediately developed two training packages: one was related to emergency management, including the COVID-19 pandemic, the other was focused on the wellbeing of human resources during crises”* (third sector, Veneto). The importance of having adequate training focused on emergency and disaster preparedness was reported as a fundamental lesson learned by most of the respondents.

#### Occupational health and safety

Several difficulties in ensuring workplace safety during the pandemic were reported, mainly due to the shortage of PPE and the inadequate IPC measures. Occupational health was also drained by the increased levels of psychological stress: “*I had to ask for psychological support because I could no longer sleep”* (primary care, Veneto). The need to guarantee HCWs' occupational safety was also perceived within NGOs. In order to improve occupational health and safety, measures aimed at enhancing PPE stockage were implemented. Conversely, little attention was paid to those measures targeting psychological wellbeing. In this regard, only one respondent reported the development of an online course targeting the wellbeing of HCWs.

#### Multisectoral and multidisciplinary collaboration

Difficulties have emerged in relation to multisectoral and multidisciplinary work. Respondents from the PHC sector described the need of a stronger collaboration with administrative staff and nurses to better manage the increased workload. Many interviewees underlined how hard it was for them to work together, especially in the presence of professionals having a different background: administrative staff, HCWs, NGO staff, etc. The main lesson learned was the need for a more concrete collaboration strategy between the administrative system and the health sector at different levels. A global health approach emerged as a successful strategy to manage the pandemic in a multidisciplinary way. Overall, the pandemic increased the awareness on the importance of interaction and collaboration between the health system and the third sector: “*The pandemic has broken a barrier: the third sector has entered the Italian national health system”* (third sector-Veneto).

### Health services delivery

The following main themes emerged when examining health services delivery: (1) public health services; (2) hospital services; and (3) primary care services.

#### Public health services

Several interviewees regarded the contact-tracing activities put in place by the public health authorities to contain the spread of the virus as inadequate: “*A total inconsistency of infection containment strategies* […] *So both from a management point of view and from a clinical point of view: zero, zero! As if it hadn't been there”* (hospital, Lombardia). The dearth of healthcare workers and a lack of coordination/integration among PHC centers and public health services were indicated as possible reasons for the weakness of the public health campaigns. The same organizational difficulties were reported in the distribution of vaccinations and the inadequacy of such health campaigns was even more relevant for vulnerable groups. Prisoners, migrants in reception centers, elderly or disabled people quarantined at home have not been adequately covered by public health initiatives. Support to these activities was granted in some contexts by local NGOs or municipalities. NGO staff and volunteers were recruited to potentiate contact-tracing activities and the distribution of vaccines in vaccination hub centers. Other initiatives involved performing COVID-19 rapid testing with the help of students or university employees, or drive-through services set up with the help of local pharmacies. Many interviewees advocated for better coordination among stakeholders in disseminating public health information to avoid conflicting messages to the population. According to the respondents, the conflicting information was a consequence of a lack of coordination among the different health authorities (i.e., national vs. regional authorities), and among politicians and mass-media. The importance of having a well-integrated and coordinated public health strategy in place emerged as one of the most important lessons learned.

#### Hospital services

Hospital services were extensively disrupted during the pandemic. The disruption concerned mostly deferrable services and resulted in the postponement of elective surgical, diagnostic procedures, or outpatient consultations. For some interviewees, the disruption of services involved even non-deferrable services, as entire hospitals were converted to management of COVID-19 cases. No measures were reported by the interviewees in response to the interruption of in-hospital services. In one case, the availability of some elective hospital services was extended to week-end days in order to reduce waiting times. According to respondents, the pandemic taught the importance of maintaining continuity of operations for all services during a disaster. Clear plans should be in place to be able to grant both deferrable and non-deferrable services for infected and non-infected patients. Another challenge that was identified by respondents concerned the level of hospital care for COVID-19 patients. Having an intermediate care unit was regarded as beneficial by respondents. This would avoid overload of intensive care units and guarantee early treatment before the development of severe forms of disease.

#### Primary care services

There is consensus among respondents that the weakness of the PHC system has been a crucial issue throughout the pandemic. Communication with PHC professionals was problematic since general practitioners were often unreachable or difficult to be tracked down. The lack of interaction between hospitals and PHC has led to sub-optimal outpatient management of both COVID-19 and non-COVID-19 patients, when discharged from hospitals. On one hand, no infrastructure was dedicated to welcoming COVID-19 cases that couldn't be discharged home (e.g., people with disabilities, people without family support at home). On the other hand, home-based care was inadequate, since no clear plans were in place for granting home-based drugs delivery, assisting with activities of daily living or rehabilitation plans for COVID-19 patients (e.g., home respiratory physiotherapy). Interviewees agree that many initiatives implemented at the community level served to sustain the PHC system and its continuity of operations. For example, additional units were established to manage COVID-19 cases at home (i.e., USCA). A pool of PHC professionals was established to grant home visits for people in need. In some cases, local pharmacies were recruited to be a reference point for disseminating health information or advice for minor health problems. Overall, the respondents agree that the pandemic showed that the PHC system is in urgent need of reform. Maintaining continuity of operations during a disaster is of paramount importance, especially for those patients who need continuous support at home.

The same challenges that were seen at the PHC level were experienced in nursing homes and community hospitals, according to interviewees. In particular, challenges were experienced in assisting COVID-19 cases inside the facilities, while concomitantly maintaining services and care for non-COVID-19 patients. In one case, reinforcing IPC measures with the help of NGO personnel allowed routine facility-based activities to be continued throughout the pandemic.

All preventative services were also postponed at the PHC level. This was identified as a challenge: “*The response to patients' health needs has worsened a lot*, [it] *has lengthened a lot because having stopped the ordinary for a very long time has led to a backlog of exams and consultations* [...]. *In order to allow patients to carry out investigations they* [PHC physicians] *have to assign higher priority to them and perhaps in that way the examination can be made in 2 or 3 months; if you respect the rules* [...] *then who knows when you will get the appointment!!”* (primary care, Piedmont). The consequences of the disruption of preventative services are not yet quantifiable.

### Logistics

The following main themes emerged when examining logistics: (1) infrastructures; (2) health supplies; (3) transports; (4) communication; (5) data management.

#### Infrastructures

Interviewees agreed with considering hospitals and other health facilities as undersized and inadequate for effective pandemic response. Paucity of hospital beds due to pre-pandemic cuts and undersized hospital departments were mentioned. Old buildings and infrastructures lacked single rooms for isolation, hampered implementation and management of the oxygen system, and rendered the delineation of dirty/clean pathways more difficult. PHC structures faced structural challenges too. As a consequence, measures were implemented to improve hospital wards and infrastructures. Spaces were reorganized and protocols were created to allow dirty/clean pathways. In one instance, a hospital was entirely dedicated to COVID-19 services. However, this caused a disruption of care for non-COVID-19 patients. New buildings and dedicated spaces were set up: external spaces for triage, COVID-19 hotels for quarantine and isolation, new infrastructures for the vaccination campaign, drive-through centers for swabs, and centers dedicated to marginalized groups: “*This quarantine and isolation center [...] was then expanded to vulnerable populations in general, because we realized that the homeless, the undocumented migrant had no access to care, and without access to care they didn't have access to COVID-19 hotels*” (third sector, Lombardia). The main lessons learned concerned the importance of structural changes in hospitals and health facilities, the importance of building hospitals with adequate space for triage and with single rooms for isolation, as well as the importance of dirty/clean pathways. Other lessons learned concerned the *containment of entrances* in hospitals, even in ordinary times, and the importance of ensuring adequate distribution of spaces to guarantee treatment for chronic patients and continuity of care.

#### Health supplies

Interviewees mentioned shortage of health and livelihood supplies as remarkable challenges faced during the pandemic. Several interviewees reported a lack and non-homogeneous distribution of medical equipment and PPE for HCWs and the population. In addition, the PPE that was available was inadequate for medical use: “*We are using PPE that is not meant for safety in the health sector* […] *these are devices for the industry, heavy and suited for a construction site, not a hospital*” (third sector, Piemonte). Measures aimed at improving the supplies' provision were: fundraising for medical supplies in hospitals, government support to PHC physicians for the supply of some equipment and PPE, and mobilization of volunteers to find equipment in other cities and regions. At the beginning of the pandemic, some kits and supplies were offered by NGOs: “*We initially used the resources of my organization because* [hospital name]*, like all the Italian hospitals, had a shortage of them. We still had the Ebola kits ready in stock, hence the first electrically ventilated gowns were ours*” (third sector, Piemonte). Notably, the NGOs that were providing support to foreign developing countries implemented measures to guarantee supplies *via* humanitarian flights. Lessons learned regarding supplies were reported by interviewees. In particular, they mentioned having learned the importance of having a detailed stockage plan, a centralized management of stockage and medical equipment, as well as the need for more research on occupational safety and adequacy of PPE.

#### Transports

Transports were disrupted during the pandemic. When hospitals were converted entirely into COVID-19 hospitals, health facilities for non COVID-19 patients were, consequently, fewer and more distant: “*From the health point of view, what was the greatest difficulty in our case was reaching the hospitals to continue the necessary therapies and to be able to attend the scheduled visits*” (policy-making, Veneto). Some solutions were implemented: transportation services managed by the municipality and/or volunteers, the offering of free public transports and free parking to health professionals, and the transportation and delivery of goods and livelihoods. Food was distributed to the elderly and people in isolation during lockdown and home delivery was implemented for drugs and medications too.

#### Communication

Different challenges concerning communication were mentioned by interviewees. Poor communication was reported among: (i) professionals and patients, or their caregivers; (ii) nursing homes and institutions; (iii) PHC physicians and hospital specialists; (iv) hospital and PHC physicians; and (v) institutions and PHC physicians. In this regard, strategies were put in place to grant communication among different levels of the health system. For example, an online platform was used to ease communication between PHC physicians, USCA and regional administration, and periodic meetings were organized at the beginning of the pandemic between USCA, regional administration and ASL. An online platform was also used to manage interaction between hospitals and PHC regarding availability of beds at the regional level. Technology has proven to be an important asset facilitating communication. For example, a system for electronic medical prescriptions and appointments was implemented and connected with pharmacies to grant continuity in the provision of medications for patients. Another type of communication problem was reported, namely the confusing and misleading information provided by the media: “*It seems to me that there was a lot of confusion. There were never exact things that were repeated by two people: one said one thing and the other said almost another thing with different nuances* […]” (patient, Piemonte). Measures were implemented to grant communication with the population. For example, the municipality communicated on a daily basis with citizens through social media platforms. Several communication channels with the population were established: dedicated email addresses, contact centers, online platforms, or door-to-door distribution of informative pamphlets. In terms of lessons learned, interviewees mentioned having learned how to use video-conferencing tools and online applications for online communication, as well as the importance of maintaining ongoing communication during disasters and health emergencies.

#### Data management

A few interviewees mentioned problems with data management during the pandemic. They referred to an overall lack of competencies in adequately collecting and storing data regarding COVID-19: “*The basic mechanisms have failed, I am still shocked today by the management of data, truly shocked. It seems to me that there is so much incompetence and inability to organize the information management system* […] *I occasionally ask how this data does not seem consistent with the other and they* [regional authorities] *never reply*” (policy-making, Lombardia).

### Changes to the H-EDRM system

Few changes to the Italian H-EDRM system were mentioned by the interviewed stakeholders. Such forward-looking strategies were deemed important for improving the response and management of future disasters. However, they do not cover all areas of the WHO H-EDRM framework (1). Changes were reported regarding *planning and coordination*. Precisely, stakeholders reported that a mechanism has been implemented to improve management and coordination of human resources and supplies (“Azienda Zero”), and that a new national center specialized in Infection Prevention and Control (IPC) has been established. Changes were reported for *human resources* too. To tackle the shortage of HCWs, the number of admissions to postgraduate medical schools has been increased. Furthermore, the stockage of PPE to improve occupational health and safety of HCWs has been advanced. With regard to *information and knowledge management*, stakeholders mentioned the establishment of digital platforms to improve communication across different areas of the health system. Among the most important changes reported by respondents is the reform of the PHC system proposed by the Italian Piano Nazionale di Ripresa e Resilienza (16), which promotes the proximity healthcare network *via* the establishment of community health infrastructures (Case della Comunità/Ospedali di Comunità). Although mentioned by almost all respondents as a much-needed reform of the Italian healthcare system, not all respondents were confident of its proper implementation at the grassroot level. The majority of the changes identified by respondents concerned the *health infrastructures and logistics*. At the hospital level, the number of beds has been reduced to be able to guarantee isolation in case of epidemics/pandemics, and “ghost wards” – namely empty departments that can be used during disasters – have been established. Funds have been allocated to Italian regions to ensure adequate provision of health supplies.

## Discussion

The COVID-19 pandemic has impacted societies, influencing countries' H-EDRM systems. By taking Italy as a case study, this research aimed to investigate the response to the COVID-19 pandemic, focusing on the changes made to the H-EDRM system, as well as the forward-looking strategies and lessons learned, by placing particular emphasis on human resources, health services delivery, and logistics. Several challenges and response strategies were mentioned by stakeholders from different sectors. From these, different lessons learned emerged, which inform changes to the way disasters and emergencies are managed in Italy. The need for pragmatic disaster preparedness plans and for structural and cultural reforms within the Italian health system were clearly pointed out. The interconnection of different sectors (e.g., PHC, hospital and third sector) was key for overcoming challenges posed by the COVID-19 pandemic.

Although Italy was a Coronavirus hotspot during the initial phase of the pandemic, there have not been many qualitative studies comprehensively exploring the Italian response to COVID-19. Qualitative studies that have been published till now examine the response from the perspective of single groups of professionals, primarily nurses (17–19) and more rarely PHC physicians (20) or specific health system components (21, 22). Since the pandemic response has implied ongoing interaction among different actors of the health system, within and beyond the medical field, and the WHO H-EDRM framework emphasizes the need for all sectors of the health system to collaborate for adequate health emergency and disaster risk management, we believe that an in-depth analysis of the multisectoral response to the COVID-19 pandemic is of great value.

As early as March 22, 2020, 4,824 HCWs were infected, and by May 4, 2020, 154 medical doctors died in Italy (23). Our findings demonstrate that inadequate training on disaster response and on the use of PPE, as well as poor occupational health and safety, were important challenges faced by HCWs and might have contributed to high infection rates among HCWs at the beginning of the pandemic. The infection of HCWs influenced the number of workers available to counteract the spread of the virus, creating a self-sustaining loop where human resources were never enough to satisfy the needs of the population. This was also due to a chronic dearth of HCWs affecting the Italian health system, together with an increased demand for care and years of cuts in public healthcare funding. Guaranteeing occupational health and safety during disasters and equipping HCWs with sufficient disaster management knowledge and competencies are key elements to ensure protection of the population. Past experiences of Middle East Respiratory Syndrome Coronavirus (MERS-CoV) and Ebola outbreaks have demonstrated that injuries and infections among first-line HCWs can be deleterious and impact the health system as a whole. Centralized management of occupational health and safety during disasters should rely on international guidelines [e.g., (24)] or specific interim guidance [e.g., (25)]. Training on the spectrum of H-EDRM capacities at all levels needs to be part of each country's disaster preparedness plan and each HCW's educational curriculum (1).

Although HCWs' psychosocial distress was pointed out as an important challenge faced during the pandemic, no long-term solution was reported by respondents in this regard. After the COVID-19 outbreak, phone-based psychological support lines were activated for HCWs in Italy. These initiatives were developed by hospitals, universities, professional associations (*e.g., Consiglio Nazionale dell'Ordine degli Psicologi, Società Psicoanalitica Italiana, Società Italiana di Terapia Comportamentale e Cognitiva)*, and the Italian Red Cross. A survey study involving a group of Italian HCWs and mental health professionals providing psychological support during the pandemic showed that, when asked if their workplace activated psychological services for employees, 29% of physicians and 20% of nurses declared that no service was activated in their facility, while 33% of physicians and 26% of nurses did not know about it (26). This shows that psychological support was not offered in such a way as to homogeneously reach all facilities and types of professionals, and that not all HCWs were aware of the availability of psychological services within their workplace. Strategies to deliver psychological support should be coordinated in a centralized way, readily available, tailored to various contexts and professional categories, and disseminated widely to reach all HCWs.

Besides the availability of psychological support for HCWs, it is reasonable to think that equipping them with adequate training and capacities, and better employment contracts and working conditions, could have prevented the psychological distress that affected them in the first place and could have decreased the chances of drop-out. Feeling physically and psychologically prepared to respond to a disaster increases the willingness of HCWs to engage in saving lives and supporting the population (27). Investing in disaster preparedness for HCWs, therefore, appears to be an effective measure to prevent several challenges that were faced during the early stages of the pandemic and can thus be a solution to improve the management of future disasters.

The lack of coordination between the public health sector and the PHC system emerged as another important challenge faced during the COVID-19 pandemic. This confirms the findings of Torri et al. (10), who reported scarce integration between the Department of Prevention and the PHC system in Italy. In line with the recommendations elaborated by Torri et al. (10), improving and enhancing integration models and tools for communication and collaboration across sectors has the potential to improve future disaster response and health system functioning as a whole.

Primary care and territorial health services were considered largely inadequate by respondents and the experience with the COVID-19 pandemic pointed out an urgent need for a PHC reform in Italy. The benefits of a more decentralized system with a stronger territorial response can be seen when looking at the case of the Veneto region, which favored proactive case and contact tracing, testing and isolation, resulting in higher rates of COVID-19 testing and home isolation, yet lower rates of hospital visits and fatalities as compared to other Italian regions (10).

By the impact observed on the Italian health system and the fragmented response across all regions, it is plausible to think that the Italian health system's preparedness plan for pandemics has been inadequate. This, coupled with the continuous financial cuts that the Italian health system has experienced in the last decades, has contributed to creating such a precarious response. This historical post-Corona moment presents the opportunity now to restructure and improve Italy's H-EDRM with a focus on a robust preparedness strategy, a fact that has vigorously emerged from this study and resonates with a vast body of literature on the same topic (28–30).

Remarkably, only few responses have been given by the interviewees of this study in respect to the impact that pandemic-related challenges and responses have had on the present and post-corona H-EDRM system. The responses were generally personal considerations rather than factual modifications based on the existing DM plans. This, together with the involvement in the study of only three Italian regions, represent important limitations of the study. This might show poor knowledge at all levels of the country's disaster management system. However, it can also indicate that the country's disaster preparedness plan has not been given enough relevance in the political agenda in the last decades, as demonstrated by the fact that the preparedness plan had not been revised in the 14 years since its development (31).

Notably, many practical measures and long-term changes to the way disasters are managed were reported regarding logistics–e.g., reduced number of hospital beds to guarantee isolation, establishment of additional wards to be able to quickly increase hospital capacity, funds to stock regional supplies of PPE and medical equipment. It is interesting to note how measures aimed at strengthening the logistics system seem to be more numerous than broader attempts of health system strengthening and reform. While it is important to define short-term measures that enhance pandemic preparedness, it is equally important to encourage complex long-term structural changes aimed at making the health system more efficient, equitable and inclusive to be able to ensure a better response to future complex emergencies with an all-hazard approach.

## Conclusions

The COVID-19 pandemic has profoundly impacted the Italian national healthcare system, prompting the implementation of diverse strategies to face the pandemic-related challenges. The main challenges and response strategies reported by stakeholders from different sectors concerned human resources, health services delivery, and logistics. From these, different lessons learned emerged and several implications for the post-pandemic Italian H-EDRM system were mentioned. Findings highlighted that the interconnection of different sectors (e.g., PHC, hospital and third sector) with a decentralized distribution of services to primary and community care was key for overcoming challenges posed by the COVID-19 pandemic. The need for pragmatic disaster preparedness plans and for structural and cultural reforms within the Italian health system were clearly pointed out as a priority to improve future disaster management.

## Data availability statement

The raw data supporting the conclusions of this article will be made available by the authors, without undue reservation.

## Author contributions

AL-C, EP, and MV designed the study, drafted the manuscript, and conducted the data collection. The drafted manuscript was revised and completed by FD, IH, and LR. All authors have approved the final version of the manuscript for submission.

## Funding

This study was conducted in collaboration with the Department of Public Health and Health Policy, Hiroshima University Japan and funded by the World Health Organization Kobe Centre for Health Development (WKC-HEDRM-K21001). The abstract of this study was accepted for digital poster display within the 2022 European Public Health (EPH) conference in Berlin.

## Conflict of interest

The authors declare that the research was conducted in the absence of any commercial or financial relationships that could be construed as a potential conflict of interest.

## Publisher's note

All claims expressed in this article are solely those of the authors and do not necessarily represent those of their affiliated organizations, or those of the publisher, the editors and the reviewers. Any product that may be evaluated in this article, or claim that may be made by its manufacturer, is not guaranteed or endorsed by the publisher.
