# Supplementary material for: The COVID-19 pandemic response and its impact on post-corona health emergency and disaster risk management in Italy
Source: Front Public Health. 2022 Oct 31;10:1034196. doi: 10.3389/fpubh.2022.1034196 (PMC9659979; doi:10.3389/fpubh.2022.1034196)
Supplement: Supplementary file 1 [file Data_Sheet_1.PDF]

INTERVIEW GUIDE
[*translated for publication purposes*]

Target group:
- Policy-level
- Hospital sector
- Primary healthcare sector
- Third sector

[*beginning of online interview*]

- Presentations round
- Presentation of the study aim and the objective of the interview
- Request for permission to audio-record the session (with clarification on anonymity and confidentiality)

[*demographic questions*]

- professional background
- years of expertise
- role during the pandemic

As anticipated, the following questions will be organized following three main areas: human resources, provision of health services and logistics.

We will now start talking about HUMAN RESOURCES.

From your perspective as a [*policymaker/hospital professional/primary care physician/third sector professional*], what are the main challenges and difficulties that your region has faced during the COVID-19 pandemic in terms of HUMAN RESOURCES?

- *Prob*: planning for staffing requirements, including surge capacity for emergency response
- *Prob*: education and training for healthcare workers in technical areas such as specialized health service delivery, incident management, epidemiology, laboratory diagnostics, information management, risk and needs assessments and logistics
- *Prob*: management of occupational health
- *Prob*: safety and security of healthcare workers

From your perspective as a [*policymaker/hospital professional/primary care physician/third sector professional*], what have been the main strategies adopted by your region to overcome the challenges you just have mentioned regarding HUMAN RESOURCES?

- *Prob*: planning for staffing requirements, including surge capacity for emergency response
- *Prob*: education and training for healthcare workers in technical areas such as specialized health service delivery, incident management, epidemiology, laboratory diagnostics, information management, risk and needs assessments and logistics
- *Prob*: management of occupational health
- *Prob*: safety and security of healthcare workers

From your perspective as a [*policymaker/hospital professional/primary care physician/third sector professional*], what do you think are the "lessons learned" after the COVID-19 pandemic regarding HUMAN RESOURCES?

- *Prob*: planning for staffing requirements, including surge capacity for emergency response
- *Prob*: education and training for healthcare workers in technical areas such as specialized health service delivery, incident management, epidemiology, laboratory diagnostics, information management, risk and needs assessments and logistics
- *Prob*: management of occupational health
- *Prob*: safety and security of healthcare workers

From your perspective as a [*policymaker/hospital professional/primary care physician/third sector professional*], what do you think are changes to the present and future Health Emergency and Disaster Risk Management system within your region regarding HUMAN RESOURCES?

- *Prob*: planning for staffing requirements, including surge capacity for emergency response
- *Prob*: education and training for healthcare workers in technical areas such as specialized health service delivery, incident management, epidemiology, laboratory diagnostics, information management, risk and needs assessments and logistics
- *Prob*: management of occupational health
- *Prob*: safety and security of healthcare workers

Now we will move on to reflect on the HEALTH SERVICES DELIVERY during the COVID-19 pandemic.

From your perspective as a [*policymaker/hospital professional/primary care physician/third sector professional*], what are the main challenges and difficulties that your region has faced during the COVID-19 pandemic in terms of HEALTH SERVICES DELIVERY?

- *Prob*: public health service
- *Prob*: pre-hospital services
- *Prob*: primary care services
- *Prob*: clinical services including emergency care, communicable disease care
- *Prob*: laboratory and diagnostic services

From your perspective as a [*policymaker/hospital professional/primary care physician/third sector professional*], what have been the main strategies adopted by your region to overcome the challenges you just have mentioned regarding HEALTH SERVICES DELIVERY?

- *Prob*: public health service
- *Prob*: pre-hospital services
- *Prob*: primary care services
- *Prob*: clinical services including emergency care, communicable disease care
- *Prob*: laboratory and diagnostic services

From your perspective as a [*policymaker/hospital professional/primary care physician/third sector professional*], what do you think are the "lessons learned" after the COVID-19 pandemic regarding HEALTH SERVICES DELIVERY?

- *Prob*: public health service
- *Prob*: pre-hospital services
- *Prob*: primary care services
- *Prob*: clinical services including emergency care, communicable disease care
- *Prob*: laboratory and diagnostic services

From your perspective as a [*policymaker/hospital professional/primary care physician/third sector professional*], what do you think are changes to the present and future Health Emergency and Disaster Risk Management system within your region regarding HEALTH SERVICES DELIVERY?

- *Prob*: public health service
- *Prob*: pre-hospital services
- *Prob*: primary care services
- *Prob*: clinical services including emergency care, communicable disease care
- *Prob*: laboratory and diagnostic services

Finally, let's move on to the last topic which is LOGISTICS.

From your perspective as a [*policymaker/hospital professional/primary care physician/third sector professional*], what are the main challenges and difficulties that your region has faced during the COVID-19 pandemic in terms of LOGISTICS?

- *Prob*: logistics systems
- *Prob*: essential supplies/medicines
- *Prob*: health emergency kits
- *Prob*: temporary health facilities
- *Prob*: stockpiling, warehousing, prepositioning of supplies
- *Prob*: transportation
- *Prob*: telecommunications
- *Prob*: security of operations
- *Prob*: donation guidelines/emergency importation of medicines

From your perspective as a [*policymaker/hospital professional/primary care physician/third sector professional*], what have been the main strategies adopted by your region to overcome the challenges you just have mentioned regarding LOGISTICS?

- *Prob*: logistics systems
- *Prob*: essential supplies/medicines
- *Prob*: health emergency kits
- *Prob*: temporary health facilities
- *Prob*: stockpiling, warehousing, prepositioning of supplies
- *Prob*: transportation
- *Prob*: telecommunications
- *Prob*: security of operations
- *Prob*: donation guidelines/emergency importation of medicines

From your perspective as a [*policymaker/hospital professional/primary care physician/third sector professional*], what do you think are the "lessons learned" after the COVID-19 pandemic regarding LOGISTICS?

- *Prob*: logistics systems
- *Prob*: essential supplies/medicines
- *Prob*: health emergency kits
- *Prob*: temporary health facilities
- *Prob*: stockpiling, warehousing, prepositioning of supplies
- *Prob*: transportation
- *Prob*: telecommunications
- *Prob*: security of operations
- *Prob*: donation guidelines/emergency importation of medicines

From your perspective as a [*policymaker/hospital professional/primary care physician/third sector professional*], what do you think are changes to the present and future Health Emergency and Disaster Risk Management system within your region regarding LOGISTICS?

- *Prob*: logistics systems
- *Prob*: essential supplies/medicines
- *Prob*: health emergency kits
- *Prob*: temporary health facilities
- *Prob*: stockpiling, warehousing, prepositioning of supplies
- *Prob*: transportation
- *Prob*: telecommunications
- *Prob*: security of operations
- *Prob*: donation guidelines/emergency importation of medicines

[*closing*]

- Ask if they have anything to add
- Thanks and greetings
