# Supplementary material for: The COVID-19 pandemic response and its impact on post-corona health emergency and disaster risk management in Italy
Source: Front Public Health. 2022 Oct 31;10:1034196. doi: 10.3389/fpubh.2022.1034196 (PMC9659979; doi:10.3389/fpubh.2022.1034196)
Supplement: Supplementary file 2 [file Data_Sheet_2.PDF]

INTERVIEW GUIDE

[*translated for publication purposes*]

Target group:

- Patients

[*beginning of online interview*]

- Presentations round
- Presentation of the study aim and the objective of the interview
- Request for permission to audio-record the session (with clarification on anonymity and confidentiality)

[*demographic questions*]

- Health profile of the patient and overview main diseases

The questions that follow revolve around the following aspects: a) main challenges and difficulties encountered during the pandemic; b) strategies implemented by your region to overcome these challenges; c) lessons that you think your region has learned following the COVID-19 pandemic; d) changes you believe will occur in the future regarding the management of disasters and emergencies.

For each of these questions, we will ask you to refer to three main topics that can guide your answer: a) human resources (e.g., doctors, nurses, health personnel with whom you interfaced during the COVID-19 pandemic); b) provision of health services; c) logistics (e.g., communication, transport).

We will start with the first question.

From your perspective, what are the main challenges and difficulties that your region has faced during the COVID-19 pandemic?

- *Prob*: human resources
- *Prob*: health services delivery
- *Prob*: logistics

From your perspective, what have been the main strategies adopted by your region to overcome the challenges you just have mentioned?

- *Prob*: human resources
- *Prob*: health services delivery
- *Prob*: logistics

From your perspective, what do you think are the “lessons learned” after the COVID-19 pandemic?

- *Prob*: human resources
- *Prob*: health services delivery
- *Prob*: logistics

From your perspective, what do you think are changes to the present and future system for the management of disasters and emergencies?

- *Prob*: human resources
- *Prob*: health services delivery
- *Prob*: logistics

[*closing*]

- Ask if they have anything to add
- Thanks and greetings
