# Supplementary material for: The COVID-19 pandemic response and its impact on post-corona health emergency and disaster risk management in Italy
Source: Front Public Health. 2022 Oct 31;10:1034196. doi: 10.3389/fpubh.2022.1034196 (PMC9659979; doi:10.3389/fpubh.2022.1034196)
Supplement: Supplementary file 3 [file Data_Sheet_3.PDF]

TABLE 1. Matrix data table reporting Italian difficulties and challenges experienced during the COVID-19 pandemic (Research Question 1) (Health professionals: HPs, Human Resources: HR; NGO: Non Governmental Organization; PPE: Personal Protective Equipment; PHC: Primary Health Care).

RESEARCH QUESTION 1<br>What difficulties and challenges has your country experienced during COVID-19?																
**REGION**		PIEDMONT					LOMBARDY					VENETO				
**PROVINCE**		Novara	Novara	Novara	Turin	Novara	Bergamo	Milan	Lodi	Milan	Milan	Padua	Padua	Padua	Padua	Padua
**INTERVIEW CODE NUMBER**		11	2	6	1	12	5	3	7	8	14	13	4	10	9	15
**SECTOR**		Policy making	Hospital	Primary Health care	Third sector	Communi ty	Policy making	Hospital	Primary Health care	Third sector	Communi ty	Policy making	Hospital	Primary Health care	Third sector	Communi ty
**HUMAN RESOURCES**																
**Management of HR**	***Shortage of HPs***		X	X	X	X	X	X	X	X	X	X	X	X	X	
	Inadequate employment contractual measures			X	X			X	X	X				X		
	Poor centralized management of healthcare workers			X	X		X	X		X		X		X		
**Education and training for competency development**	***Inadequate training***		X		X		X	X	X	X			X	X	X	
	Quickly changing guidelines and protocols									X				X		
**Occupational health and safety**	***Shortage of PPE***		X	X	X		X		X	X				X	X	
	Psychological stress/burnout of HPs				X				X	X			X	X	X	
	Protect safety of HPs (concerning NGOs)														X	
	Poor infection prevention and control measures										X			X	X	
**Multisectoral and multidisciplinary collaboration**	***Problems with multidisciplinary work***					X		X		X				X	X	
**HEALTH SERVICES DELIVERY**																

Public Health Services	*Weakness of health campaigns*							X						X	X	
	Interruption of public health services for non COVID-19 patients			X						X						
	Unsystematic information management and risk communications	X				X	X		X		X	X				X
	Weakness of policies, strategies and legislations to support citizens								X	X	X	X				X
Hospital Services	*Prolonged interruptions of deferrable services both for COVID-19 and non COVID-19 patients*		X		X	X			X		X		X	X		X
	Prolonged interruptions of non deferrable services for non COVID-19 patients											X	X			
	Inadequacy of patients' intra-hospital management												X		X	
Primary Care Services	*Weak PHC*	X	X	X	X		X	X			X		X			X
	Lack of extra - hospital services for Sars-CoV2 positive patients			X												
	Nursing homes and community hospitals crisis		X						X						X	
HEALTH INFRASTRUCTURES AND LOGISTIC																
Safe infrastructures	*Undersized/Inadequate hospital wards*	X	X		X			X			X			X	X	
	Inadequate primary care facilities													X		
Supplies	*Availability and adequacy of health supplies*		X	X	X		X		X	X	X		X		X	
	Shortage of livelihoods supplies											X				

	High costs for buying emergency equipment and supplies (for PHC physicians)													X		
**Transportation**	***Lack of transportation services***			X								X				
**Communication**	***Problems in communication***			X					X					X		V
	Poor risk communication strategy					X						X				
**Data management**	***Mismatching data across different databases***						X									
	Lack of adequate software for collecting data							X								
