# Supplementary material for: The COVID-19 pandemic response and its impact on post-corona health emergency and disaster risk management in Italy
Source: Front Public Health. 2022 Oct 31;10:1034196. doi: 10.3389/fpubh.2022.1034196 (PMC9659979; doi:10.3389/fpubh.2022.1034196)
Supplement: Supplementary file 4 [file Data_Sheet_4.PDF]

TABLE 2. Matrix data table reporting Italian Responses to the difficulties and challenges experienced during the COVID-19 pandemic (Research Question 2) (Health professionals: HPs, Human Resources: HR; NGO: Non Governmental Organization; PPE: Personal Protective Equipment; PHC: Primary Health Care).

.

RESEARCH QUESTION 2<br>How Did Your Country Respond To The Challenges that you Mentioned?																
**REGION**		**PIEDMONT**					**LOMBARDY**					**VENETO**				
**PROVINCE**		Novara	Novara	Novara	Torino	Novara	Bergamo	Milano	Lodi	Milano	Milano	Padova	Padova	Padova	Padova	Padova
**INTERVIEW CODE NUMBER**		11	2	6	1	12	5	3	7	8	14	13	4	10	9	15
**SECTOR**		Policy making	Hospital	Primary Health care	Third sector	Communi ty	Policy making	Hospital	Primary Health care	Third sector	Communi ty	Policy making	Hospital	Primary Health care	Third sector	Communi ty
**HUMAN RESOURCES**																
**Management of HR**	***Recruitment of extra-HR***	X	X	X	X		X	X	X	X		X	X	X	X	
	Support form NGOs									X					X	
	Volunteers recruitment	X					X					X			X	
**Education and training for competency development**	***Improvement of training***		X	X	X				X				X		X	X
	Dissemination and sharing of guidelines and protocols			X										X		
**Occupational health and safety**	***Implementation of PPE stockage***		X		X		X		X						X	
	Implementation of Infection Prevention and control measures												X	X		
	Measures to increase the awareness concerning the well being of health care workers														X	
**Multisectoral and multidisciplinary collaboration**	***Integration and coordination***	X					X		X	X		X			X	
**HEALTH SERVICES DELIVERY**																
**Public Health Services**	***Initiatives to support vulnerable populations***	X			X					X		X				

	Strengthening of vaccination campaigns	X										X			X	
	Strengthening of Infection Prevention and Control measures	X								X		X			X	
	Policies and legislations	X					X				X					X
**Hospital Services**	*Recovering of interrupted deferrable services*			X							X					
**Primary Care Services**	***Strengthening of PHC***		X	X	X			X								X
	Strengthening of Nursing homes								X	X						
**HEALTH INFRASTRUCTURES AND LOGISTIC**																
**Safe infrastructures**	***Improvement of hospital wards***		X	X				X	X		X		X			
	Creation of new buildings/dedicated spaces and structures	X	X	X					X						X	
**Supplies**	***Improvement of supplies provision***	X			X		X		X			X			X	
**Transportation**	***Implementation of transportation services***	X					X					X				
	Transportation/delivery of goods	X			X						X	X				
**Communication**	***Implementation of Communication services***	X	X	X			X		X							X
	Use of technology	X			X	X						X				X
