# Supplementary material for: The COVID-19 pandemic response and its impact on post-corona health emergency and disaster risk management in Italy
Source: Front Public Health. 2022 Oct 31;10:1034196. doi: 10.3389/fpubh.2022.1034196 (PMC9659979; doi:10.3389/fpubh.2022.1034196)
Supplement: Supplementary file 5 [file Data_Sheet_5.PDF]

TABLE 3. Matrix data table reporting Italian lessons learnt from the COVID-19 pandemic to prepare for future response (Research Question 3) (Human Resources: HR; NGO: Non-Governmental Organization; PPE: Personal Protective Equipment; PHC: Primary Health Care, USCA: Special Unit for the Continuity of Care).

RESEARCH QUESTION 3<br>What did you learn from the current COVID-19 event to prepare for the future response?																
**REGION**		**PIEDMONT**					**LOMBARDY**					**VENETO**				
**PROVINCE**		Novara	Novara	Novara	Torino	Novara	Bergamo	Milano	Lodi	Milano	Milano	Padova	Padova	Padova	Padova	Padova
**INTERVIEW CODE NUMBER**		11	2	6	1	12	5	3	7	8	14	13	4	10	9	15
**SECTOR**		Policy making	Hospital	Primary Health care	Third sector	Communi ty	Policy making	Hospital	Primary Health care	Third sector	Communi ty	Policy making	Hospital	Primary Health care	Third sector	Communi ty
**HUMAN RESOURCES**																
**Management of HR**	***The importance of increasing HR***		X	X	X	X	X			X		X	X	X	X	
	The importance of volunteers recruitment											X			X	
	The importance of adequate employment contractual measures		X		X				X							
	The importance of having a centralized management of healthcare workers							X				X		X		
**Education and training for competency development**	***The importance of an adequate training***		X		X					X		X	X	X	X	X
	The importance of sharing updated guidelines and protocols												X	X	X	
**Occupational health and safety**	***The importance to guarantee safety of health care worker***				X											
	The importance of ensuring healthcare workers well-being														X	

**Multisectoral and multidisciplinary collaboration**	***The importance of multidisciplinarity***	X					X	X	X	X		X		X	X	
	The importance of collaboration with Non Governmental Organizations (NGOs)									X					X	
**HEALTH SERVICES DELIVERY**																
**Public Health Services**	***The importance of having an adequate vaccination campaign***															X
	The importance of strengthening Infection and Prevention control measures								X							
	The importance of information management and risk communications								X		X					X
	The importance of policies, strategies and legislations	X					X				X	X			X	X
**Hospital Services**	***The importance of maintaining all the health services during a pandemic disaster***					X			X		X	X	X			X
**Primary Care Services**	***Improving PHC***	X	X	X	X			X		X			X			
	Maintaining home health services both for COVID-19 and non COVID-19 patients															X
	USCA model for continuity of care			X												
	Adequate distribution of health care services					X										
**HEALTH INFRASTRUCTURES AND LOGISTIC**																
**Safe infrastructures**	***Importance of structural changes in hospitals/health care facilities***		X		X			X					X		X	
	Containment of overall entrances in hospitals		X													

	No more conversion of hospitals into COVID-19 hospitals exclusively											X				
**Supplies**	*The importance of having an adequate stockage of supplies*				X				X				X		X	
	Importance of centralized management of stockage of health supplies and medical equipment						X						X			
	More research on occupational safety and adequacy of PPE				X											
**Communication**	*Progress in use of videoconferencing tools and online applications*	X		X							X	X				
	Importance of maintaining ongoing communication during disasters/emergencies								X							
