# Supplementary material for: The COVID-19 pandemic response and its impact on post-corona health emergency and disaster risk management in Italy
Source: Front Public Health. 2022 Oct 31;10:1034196. doi: 10.3389/fpubh.2022.1034196 (PMC9659979; doi:10.3389/fpubh.2022.1034196)
Supplement: Supplementary file 6 [file Data_Sheet_6.PDF]

TABLE 4. Matrix data table reporting the influence of the Italian pandemic challenges and responses on the present system and post-corona H-EDRM system (Research question 4) (Human Resources: HR; PPE: Personal Protective Equipment; PHC: Primary Health Care).

RESEARCH QUESTION 4
What influence have these challenges and responses had on the present system and post-corona H-EDRM system?

REGION		PIEDMONT					LOMBARDY					VENETO				
PROVINCE		Novara	Novara	Novara	Turin	Novara	Bergamo	Milan	Lodi	Milan	Milan	Padua	Padua	Padua	Padua	Padua
INTERVIEW CODE NUMBER		11	2	6	1	12	5	3	7	8	14	13	4	10	9	15
SECTOR		Policy making	Hospital	Primary Health care	Third sector	Commun ity	Policy making	Hospital	Primary Health care	Third sector	Commun ity	Policy making	Hospital	Primary Health care	Third sector	Commun ity
**HUMAN RESOURCES**																
Management of HR	*Centralized management of HR*								X			X	X	X		
	Contractual measures improvement													X		
	Measures to increase the number of healthcare workers				X				X							
**HEALTH SERVICES DELIVERY**																
Public Health Services	*Improved prevention and control of infectious diseases*							X	X							
	Policies, strategies and legislations										X					X
Hospital services	*Research improvement (establishment of a new National Center for Infection Prevention and Control)*							X								
Primary Care Services	*PHC reform ("Piano Nazionale di Ripresa e Resilienza")*		X		X		X							X		
**HEALTH INFRASTRUCTURES AND LOGISTICS**																
Infrastructures	*Prepared infrastructures*	X	X					X						X		
Supplies	*Measures to guarantee supplies in case of disaster*	X			X			X							X	
Communication	*Digital advancement*		X													

	Communication channels		X	X												
